# Supplementary material for: MetaQTL: a package of new computational methods for the meta-analysis of QTL mapping experiments
Source: BMC Bioinformatics. 2007 Feb 8;8:49. doi: 10.1186/1471-2105-8-49 (PMC1808479; doi:10.1186/1471-2105-8-49)
Supplement: Additional File 7 — MetaQTL Package : jar file and tutorial. This Zip archive contains both the MetaQTL JAR file and the files of the tutorial. [file 1471-2105-8-49-S7.zip › org.inra.metaqtl/doc/org/thalia/bio/entity/adapter/package-tree.html]

org.thalia.bio.entity.adapter Class Hierarchy


|  |  |  |
| --- | --- | --- |
| **Overview** \| **Package** \| Class \| Use \| **Tree** \| **Deprecated** \| **Index** \| **Help** | |  |
| **PREV**   **NEXT** | **FRAMES**    **NO FRAMES**     **All Classes** |  |


---


## Hierarchy For Package org.thalia.bio.entity.adapter

**Package Hierarchies:**: All Packages

---

## Class Hierarchy

- java.lang.Object
  - org.thalia.bio.entity.adapter.**BioEntityAdapter** (implements org.thalia.bio.IBioAdapter)
    - org.thalia.bio.entity.adapter.**BioEntityCollectionAdapter** (implements org.thalia.bio.IBioAdapter)- org.thalia.bio.entity.adapter.**GeneticMapBeanAdapter** (implements org.thalia.bio.IBioAdapter)- org.thalia.bio.entity.adapter.**LGroupBeanAdapter** (implements org.thalia.bio.IBioAdapter)- org.thalia.bio.entity.adapter.**LocusBeanAdapter**- org.thalia.bio.entity.adapter.**OntologyBeanAdapter**- org.thalia.bio.entity.adapter.**OntologyTermBeanAdapter**

---


|  |  |  |
| --- | --- | --- |
| **Overview** \| **Package** \| Class \| Use \| **Tree** \| **Deprecated** \| **Index** \| **Help** | |  |
| **PREV**   **NEXT** | **FRAMES**    **NO FRAMES**     **All Classes** |  |


---
